# Sound iconicity of abstract concepts: Place of articulation is implicitly associated with abstract concepts of size and social dominance

**DOI:** 10.1371/journal.pone.0187196

**Published:** 2017-11-01

**Authors:** Jan Auracher

**Affiliations:** Department for Language and Literature, Max Planck Institute for Empirical Aesthetics, Frankfurt aM, Germany; Kyoto University, JAPAN

## Abstract

The concept of sound iconicity implies that phonemes are intrinsically associated with non-acoustic phenomena, such as emotional expression, object size or shape, or other perceptual features. In this respect, sound iconicity is related to other forms of cross-modal associations in which stimuli from different sensory modalities are associated with each other due to the implicitly perceived correspondence of their primal features. One prominent example is the association between vowels, categorized according to their place of articulation, and size, with back vowels being associated with bigness and front vowels with smallness. However, to date the relative influence of perceptual and conceptual cognitive processing on this association is not clear. To bridge this gap, three experiments were conducted in which associations between nonsense words and pictures of animals or emotional body postures were tested. In these experiments participants had to infer the relation between visual stimuli and the notion of size from the content of the pictures, while directly perceivable features did not support–or even contradicted–the predicted association. Results show that implicit associations between articulatory-acoustic characteristics of phonemes and pictures are mainly influenced by semantic features, i.e., the content of a picture, whereas the influence of perceivable features, i.e., size or shape, is overridden. This suggests that abstract semantic concepts can function as an interface between different sensory modalities, facilitating cross-modal associations.

## Introduction

How do we convey meaning in verbal interaction? In contrast to mainstream linguistics, the field of phonosemantics claims that phonemes themselves carry inherent semantic information. Within phonosemantics, sound iconicity is defined as an acoustic representation of non-acoustic phenomena in a non-arbitrary manner [1]. Sound iconicity has been referred to by various terms, most prominently by Hinton and colleagues, who use the term *synaesthetic sound iconicity* [2]. However, this term is misleading for two reasons: First, *synaesthesia* usually refers to a phenomenon that is restricted to specific individuals and, second, the attribute *symbolic* implies a relation between sound and meaning that is based on convention. By contrast, an extensive body of literature provides solid evidence suggesting that sound iconicity is truly iconic and universal insofar as it is based on perceived associations of articulatory-acoustic features of phonemes with non-acoustic characteristics, such as size, taste, or emotional valence, and as these associations have been found across languages and cultures (for reviews and discussions of the terminology, see [2, 3–8]).

Recent research emphasized the functionality of sound iconicity, facilitating, in particular, word learning and communication (for an overview see [9]). Studies, for example, found that Japanese words in which sound and meaning correspond with each other are easier to memorize for native speakers of English [10, 11] and of Dutch [12] when compared to words with an arbitrary relation between sound and meaning. Interestingly, the same seems to hold true for first language acquisition. Several studies report a significant higher likelihood for sound iconic words to be learned in an early stage of language acquisition than for non-iconic words [13–16]. Yet, another line of research has focused on the communicative role of emotional connotations elicited by sound iconicity. Shrum and colleagues [17], for example, examined how sound iconicity can be used in brand names to communicate (alleged) attributes of products (see also [18, 19]). Other studies have found evidence suggesting that sound iconicity is used in poetic language to convey the emotional tone of poems and song lyrics [1, 20–23].

Though there is a long tradition of behavioral, developmental, corpus-linguistic, and (more recently) neurocognitive studies on sound iconicity, the vast majority of these studies have been dedicated to the question of whether sound iconicity exists in the first place, and the focus has only recently shifted towards a better understanding of the cognitive processes underlying sound iconicity [8]. For example, EEG studies analyzing event-related potentials during the processing of words with or without sound iconicity have found evidence suggesting that sound-iconic words can cause a co-activation of otherwise modality-specific areas in the brain during early sensory processing [24–26]. On the other hand, studies applying brain imaging techniques (fMRI) have found increased activity in fields that had previously been related to multi-modal integration [27, 28]. However, while these studies are critical to the establishment of a better understanding of neurocognitive processes related to sound iconicity, they still leave pivotal questions unanswered. For one, there has been some–partly controversial–discussion about the role of abstract conceptual thinking in cross-modal associations [29–34]. The association between luminance and sound pitch, for example, could be due to a perceived correspondence at the level of physical characteristics (linking the intensity of light directly with the frequency of sound) or at the level of semantic connotations (e.g., mediated by a shared hedonic value) [35]. In fact, scholars such as Hornbostel have suggested the existence of an amodal concept of brightness that links sensation across different sensory modalities [36].

It is important to note, however, that perceptual and conceptual processing do not mutually exclude each other as sources of sound iconicity. On the contrary, it is likely that both forms are involved at different stages of the processing of sound-iconic words. However, the studies outlined above call into question which role sensory analogy and semantic analogy play between the acoustic appearance of a word and its meaning in sound-iconic associations. The aim of the current paper will be to compare the influence of directly perceivable features (such as size) with the influence of abstract, semantic features (such as content) on the association of visual stimuli with vowels, differing in their place of articulation.

One of the most-studied examples of sound iconicity is the relationship between articulatory characteristics of vowels and their association with size (for reviews see [3, 6, 37–39]). In an early study, Sapir [40] found that the association of nonsense syllables, consisting of consonant-vowel-consonant combinations, with either small or big objects is determined by the central vowel of the syllables. Newman [41], who conducted a follow-up study, concluded that specific acoustic and articulatory characteristics determine the association of vowels with size, claiming that vowels with high pitch and smaller vocal cavity are preferably associated with stimuli related to smallness, while vowels with low pitch and larger vocal cavity are preferably associated with stimuli related to bigness.

Later studies asked participants to assess vowels–single or in combinations with consonants–on a list of bi-polar items, such as big-small, fast-slow, bright-dark, or good-bad [42–46]. An extensive discussion of their results would clearly overstretch this introduction, however, three major findings shall be highlighted here. First, Greenberg and Jenkins [42], applying a paper-and-pencil test, found that the place of articulation of vowels correlates with their assessment on items referring to size (see also [43, 44, 45]). That is, vowels that are articulated toward the back of the vocal tract are more readily associated with attributes expressing bigness, and as articulation moves toward the front of the articulatory tract, association with ‘small’ and related attributes increases. Second, this association has been found not only for size but also for related concepts referring to physical or social dominance, such as *strong-weak* [42] or *powerful-powerless* [45]. And third: the relation between articulatory characteristics of phonemes and their association with size seems to be universal, as it has been found independent of participants’ native language [44, 45, 46]. In fact, studies examining a wide range of world languages found that front vowels occur with an unusual high frequency in words and morphemes that express smallness and associated categories [47, 48]. Moreover, Peña et al. [49] reported that even four-month-old infants show similar preferences to match the place of articulation of vowels with object size.

In an attempt to explain the association between place of articulation and size, it has been related to the so called size-pitch effect, according to which low-pitch sounds are associated with bigness and high-pitched sounds are associated with smallness [33, 39, 50]. This is insofar interesting as research suggests that lower pitch is not only associated with directly perceivable size but also with semantic stimuli (i.e., words) that denote size or related concepts, such as weight or thickness [51]. Moreover, Eitan and Timmers [52] reported that participants when asked tend to match low pitch to words that are related to bigness such as attributes like “thick, heavy, strong, and male” or objects like “crocodile”, while the opposite holds for high pitch. Finally, evidence from studies of animal behavior indicates that in many animal species, the voice pitch of males predicts their social status and mating success [53–55]. Similarly, studies of human verbal interaction revealed that voice pitch is a good indicator of a male’s attractiveness for females, as well as his–actual or alleged–physical or social dominance [56–62].

To sum up, there is sound evidence suggesting that pitch is not only related to size but more to an abstract concept of physical or social dominance. According to Ohala [63], this association of pitch has found its way into human language, causing a universal association that connects back vowels with bigness, strength, or high-dominance and front vowels with smallness, weakness, or low-dominance. Following this claim, one would expect that the articulatory place of vowels allows to convey meaning on an abstract conceptual level. Consequently, it was hypothesized that sound iconic associations can also be found with non-acoustic stimuli that refer to size or related concepts on a semantic level, e.g. through the content of a picture. In the following, three experiments designed to test this hypothesis will be outlined. The aim of these experiments was to show that sound iconic associations between the place of articulation and size are not (necessarily) triggered by directly perceivable properties, such as the size of a visual stimulus or the denotation of a word, but can also be triggered by abstract conceptual properties, such as the inferences about the size of a depicted object or the relation between behavior and social status.

## Research objective and design

The objective of the current study was to investigate whether sound iconicity of magnitude enables the prediction of implicit associations of pseudo-words with visual stimuli depicting physical or social dominance. By pseudo-words, I mean consonant-vowel combinations that are phonologically possible but have no lexical denotation in a specific language. Following the frequency code hypothesis by Ohala [50, 63], I hypothesized that pictures depicting big, strong, aggressive or dominant objects or behavior would be associated with pseudo-words containing back vowels, while pictures depicting small, weak, fearful, or submissive objects or behavior would be associated with pseudo-words containing front vowels.

**Fig 1:**
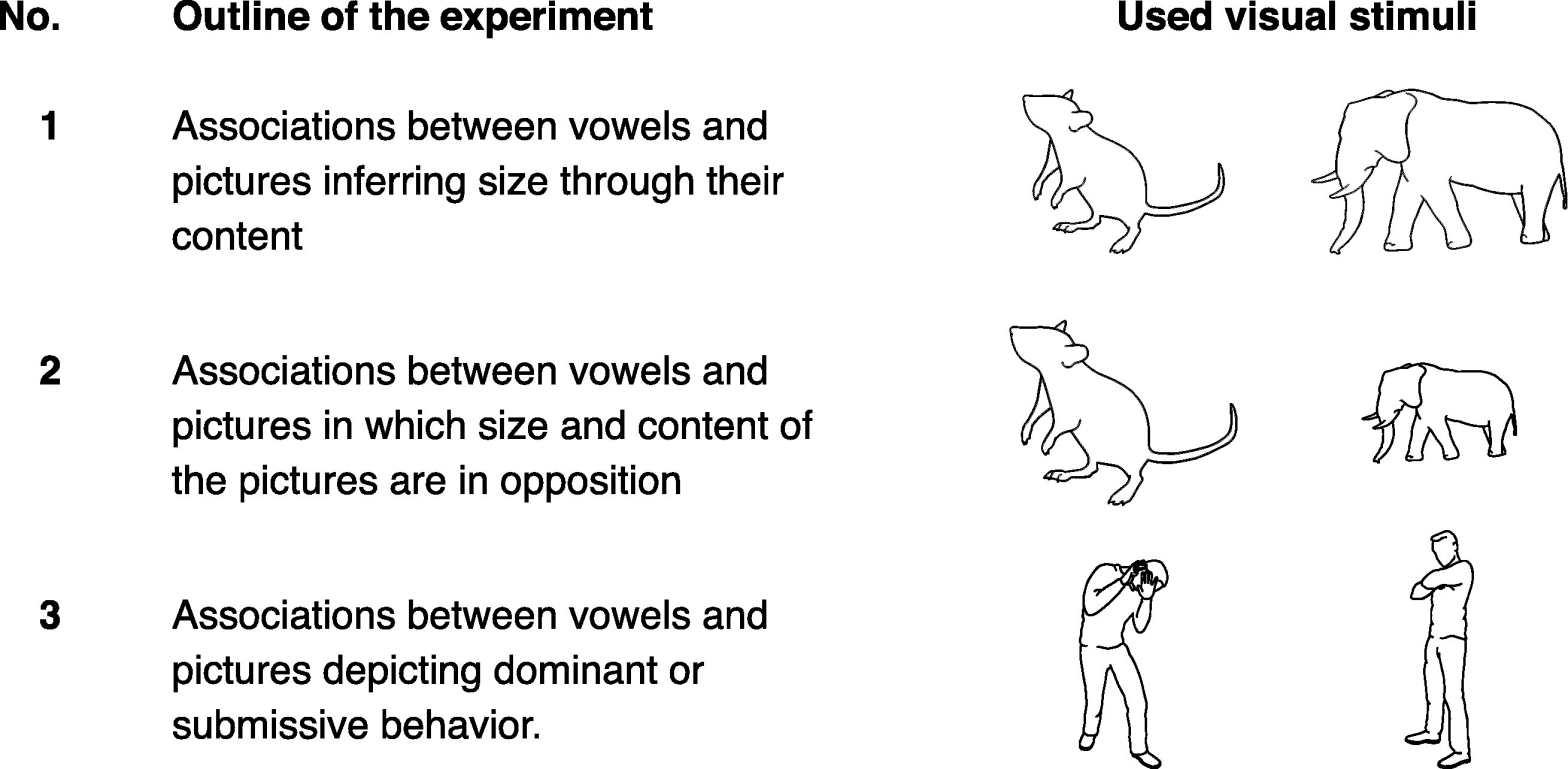
Overview of experiments with examples of visual stimuli. Visual stimuli in the left column were expected to be associated with front vowels; pictures in the right column were expected to be associated with back vowels.

In the first experiment, visual stimuli consisted of pictures depicting either small or big animals, whereas the pictures themselves were of the same size. Thus, unlike previous experiments, in which visual stimuli directly embodied the notion of either bigness or smallness (e.g., through the size of a picture or the meaning of a word), in this experiment the relation between the stimuli and the concept of size relied on participants’ interpretation of stimulus content. The aim of this experiment, therefore, was to test the relation between pitch and size using stimuli that imply a difference in size without making it explicit. In the second experiment, the same pictures were used, however, this time the pictures depicting small animals were larger than the pictures depicting big animals. That is, perceptible features of the pictures were incongruent with their content. Thus, the second experiment targeted on the interaction between perceptible and semantic features in sound iconic associations. Assuming that sound iconicity is based on semantic connotations elicited by acoustic characteristics of phonemes, it was hypothesized that the size of the picture should show only a negligible effect on the association between phonemes and the content of the pictures. In the last experiment, pictures depicting either dominant or submissive body postures were used as visual stimuli. Thus, in this experiment it was tested whether the association between pitch and size can also be extended to social behavior. It was assumed that back vowels are associated with behavior perceived as dominant while, vice versa, front vowels are associated with behavior perceived as submissive. The three experiments are summarized in Fig 1.

## Experiment I

The first experiment was conducted to see whether the relationship between the place of articulation of vowels and size can also be found with semantic features that imply rather than depict or denote respective concepts. To this end, pseudo-words containing back or front vowels were tested for implicit associations with pictures of big and small animals. Like experiments 2 and 3, experiment 1 was conducted in Japan; thus, all instructions were written in Japanese.

### Method

#### Participants

The participants were 30 university students (27 female) aged between 18 and 21 (mean 19). All participants were native Japanese speakers who reported no hearing impairments and had normal or corrected-to-normal vision. Participation was voluntary and participants received a 500-yen gift certificate. All participants gave written informed consent to participate in the study. The experiments were approved by the Ethics Committee of Doshisha University.

#### Materials

**Fig 2:**
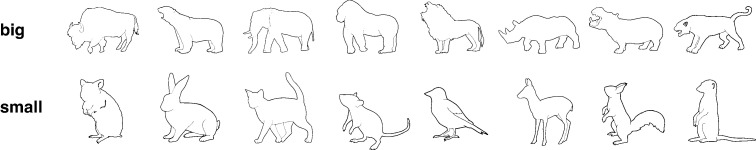
Visual stimuli showing either big or small animals.

For visual stimuli sixteen pictures of either big or small animals were used (Fig 2). The animal pictures were drawn by a professional illustrator (NiKo-Illustration). The illustrator was instructed to draw either “big, strong, and heavy” or “small, weak, and light” animals. Otherwise the illustrator was uninformed with regard to the goal of the research. All animals were depicted in a three-quarter side view facing to the left.

Two groups of pseudo-words were generated by randomly creating sequences of three syllables, each consisting of one consonant and one vowel (CVCVCV). The two groups differed with respect to their vowels; while the first group contained back vowels (/o/ and /u/), the second group contained front vowels (/i/ and /e/). To avoid the influence of the consonants, the plosive consonants /p/, /t/, and /k/ were used for both groups of pseudo-words.

Eight pseudo-words per group were recorded by a male speaker (pseudo-words with back vowels: kotopu, kutopo, kopotu, pokotu, putoko, tokopu, tupuko, topuko; pseudo-words with front vowels: kipite, kitepi, pekite, tipeki, pikite, piteke, tepeki, tikipi). The recordings were done in an anechoic room, using a Roland CD-2e digital recorder. The speaker was asked to clearly put the stress on the first syllable when pronouncing each word. Otherwise he was uninformed with respect to the aim of the experiment. The length of the stimuli was manipulated to fit exactly 0.58 sec, using Audacity version 2.0. Files were saved in WAV 16 Bit PCM format.

In order to exclude undesired effects caused by phonetic congruence between the (Japanese) names of the depicted animals and the phonetic characteristics of the pseudo-words, precautions were taken by (1) keeping the number of front-vowels and back-vowels balanced within each group (i.e., small animals and big animals), (2) minimizing the number of consonant-vowel combinations that occur in the acoustic stimuli (i.e., plosive + front-vowel or plosive + back-vowel), (3) preferably selecting animals with Japanese names that contain both front-vowels and back-vowels, and (4) apportioning the number of animals that contain only front-vowels or only back-vowels within each group. The Japanese names were collected in a pre-study in which 12 participants were asked to provide name(s) that spontaneously came to mind when looking at the picture. The Japanese names are listed below in Roman letters (for Katakana, see supporting materials S1 Table). Answers that were given by a majority of participants are listed outside the brackets. Participants also provided alternative answers for some animals, which are listed inside the brackets. The order of animals is consistent with their occurrence in Fig 2 from left to right and from top to bottom: BISON: ushi (bison, baffarō, gnū); POLAR BEAR: shirokuma (kuma); ELEPHANT: zō; GORILLA: gorira; LION: raion; RHINO: sai; HIPPO: kaba; CHEETAH: chītā (hyou, tora); HAMSTER: hamustā (nezumi, morumotto, momonga); RABBIT: usagi; CAT: neko (inu); MOUSE: nezumi; BIRD: tori (hato, inko); DEER: shika (banbi, inpara); SQUIRRIL: risu; MEERKAT: mīakyatto (pureridogu, okojo). Based on the answers given by a relative majority of participants, the distribution of front-vowels and back-vowels is as follows:

- Ratio of front-vowels to back-vowels per group: small animals: 8/8, big animals: 6/6.
- Ratio of consonant-vowel combinations that also occur in the acoustic stimuli (consonant + front-vowel vs. consonant + back-vowel): small animals: 0/3, big animals: 0/1.
- Number of animals with both front-vowels and back-vowels in their Japanese names: small animals: 6, big animals: 4.
- Ratio of animals with only front-vowels vs. animals with only back-vowels: Small animals: 1/1, big animals: 2/1.

To summarize, the ratio of front-vowels to back-vowels was balanced for both groups of animals. Unequal distributions regarding consonant-vowel combinations and the number of animals with only front-vowels or only back-vowels predicted a result that contradicted the hypothesis (i.e., more consonant + back-vowel combinations among small animals and more names with only front-vowels in the group with big animals). Thus, potential confounding variables based on phonetic congruency between visual and acoustic stimuli were excluded as far as possible.

#### Apparatus

The experiments were conducted in a quiet atmosphere free from disturbing noise or other distractions. Visual stimuli were presented on a 15.6” HD LED LCD display. Acoustic stimuli were presented using a Sony MDR-CD900ST headphone. During presentation of the acoustic stimuli, a white fixation cross on black background appeared on the screen. For stimulus delivery and experimental control, the software Presentation® (version 15) by Neuro-Behavioral-Systems (http://www.neurobs.com/) was used.

#### Procedure

The hypothesis was tested using the Implicit Association Test (IAT) [64]. According to Parise and Spence [65], who used the IAT in a study of cross-modal associations, the design of this test has the advantages of (a) being independent of participants’ ability or willingness to accurately report their introspection, (b) avoiding the risk of merely assessing failure of selective attention on the part of the participant, and (c) allowing for the measurement of mutual associations, rather than assessing influence in a single direction from one modality to another.

In the current research, participants were presented with two categories of pseudo-words and two categories of pictures. Stimuli of both modalities–acoustic and visual–were presented in random order. Participants were asked to categorize each stimulus by pressing a button with either the left or the right index finger. The Implicit Association Test is based on the idea that participants encounter fewer problems and hence are able to complete the task faster and with fewer mistakes when associated stimuli–e.g., visual stimuli related to bigness and acoustic stimuli with low frequency–are allocated to the same response behavior (i.e., pressing buttons on the same side) than when they are allocated to different response behaviors (i.e., pressing buttons on opposite sides). Each participant performed two experimental blocks, one in which presumably associated groups of stimuli were allocated to the same side (conforming condition) and one in which presumably associated groups of stimuli were allocated to opposite sides (non-conforming condition). The hypothesis was corroborated when response latency in the conforming condition was significantly shorter than that in the non-conforming condition.

**Fig 3:**
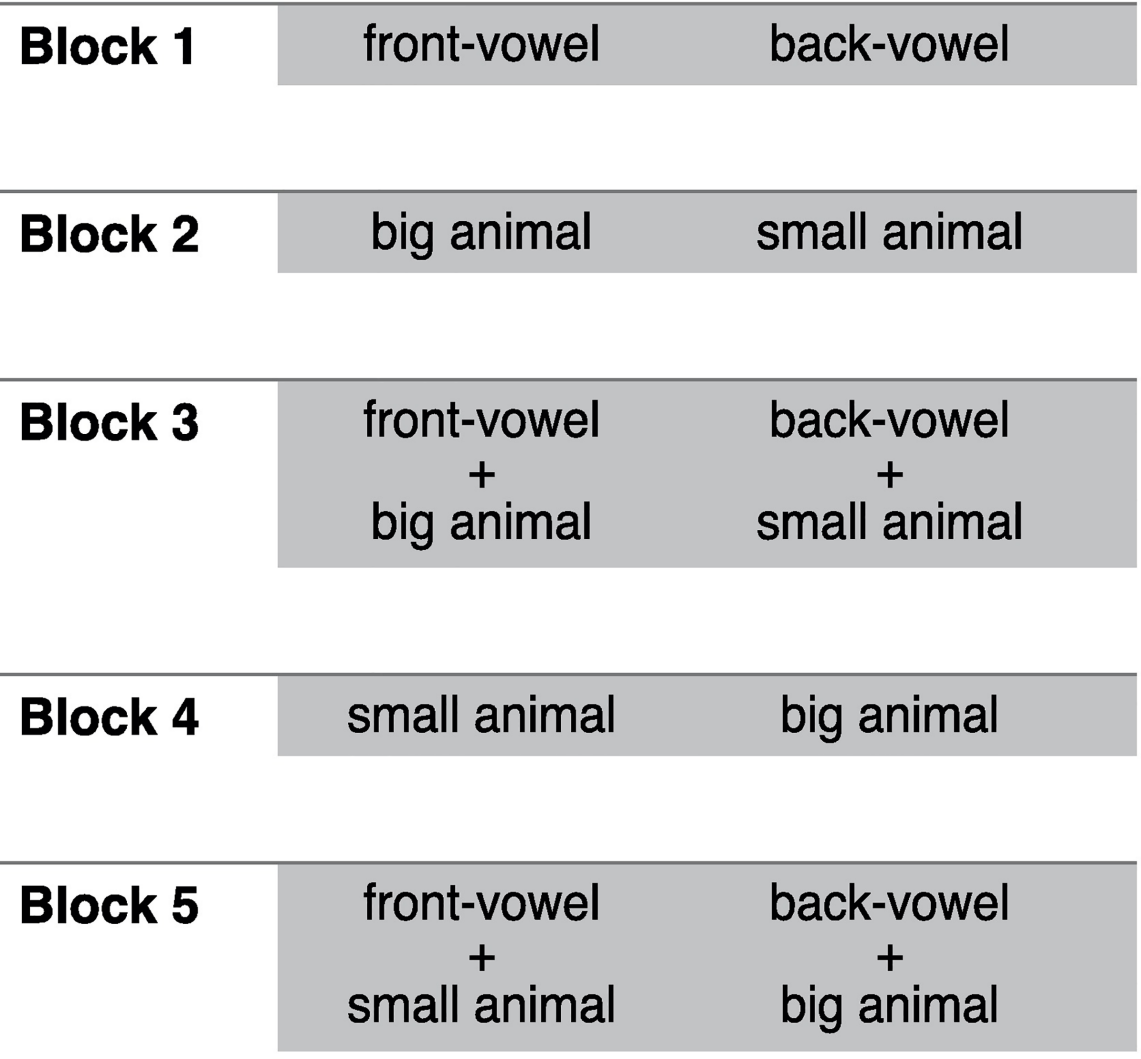
Experimental design. IAT in five blocks, with blocks 1, 2, and 4 for training and blocks 3 and 5 for the experiment. In the experiment, *big animals* were labeled as “wild animals”, small animals as “gentle animals”, front vowels as “bright sounds”, and back vowels as “dark sounds”. Stimuli on the left-hand side were categorized by pressing the [E] key. Stimuli on the right-hand side were categorized by pressing the [I] key. The figure shows an example for a possible sequence. The order of the two experimental blocks as well as the allocation of the stimuli to either the right or the left side was randomized.

Following the standard procedure of the IAT [66], each experiment consisted of five blocks in total: three for training and the other two for the experiment (Fig 3). In the training blocks–numbers 1, 2, and 4 –participants practiced allocation of the stimuli for each modality separately (e.g., first for pseudo-words and second for pictures). In the experimental blocks–numbers 3 and 5 –stimuli of both dimensions were presented together in randomized order. In block 4, the allocation of the acoustic stimuli changed sides. The order of the two experimental blocks (e.g., first conforming and second non-conforming condition) and the allocation of the stimuli (e.g., big animals to the right and small animals to the left in conforming block) was randomized.

Participants first read an introduction which explained the procedure and introduced the stimuli (for the original introduction together with an English translation see S1 Text). To make sure that participants were not primed to focus on differences in size, animals were introduced as being either wild (big animals) or gentle (small animals). In Japanese, the expressions read 獰猛な動物 for wild (big) and 温和な動物 for gentle (small) animals. However, to avoid confusion I will stick to the distinction between ‘big’ and ‘small’ animals in what follows. Pseudo-words with back- and front vowels were introduced as 暗い音 (dark sounds) and 明るい音 (bright sounds), respectively. After the introduction, the allocation of the stimuli to either the left or the right side was displayed, followed by the training sessions.

**Fig 4:**
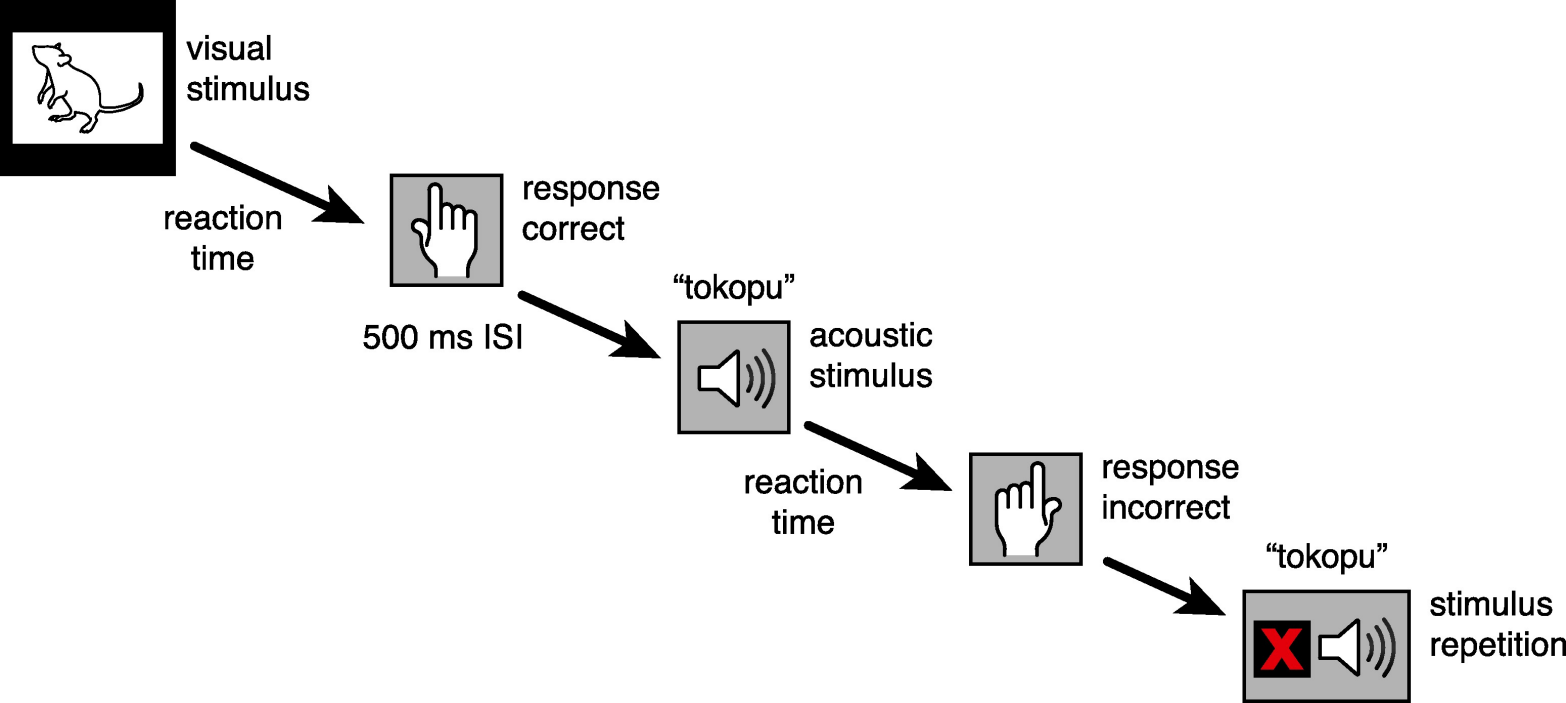
Trial design. The figure shows an example for a sequence taken from block 3 (see Fig 3). Incorrect answers were signaled by a repetition of the same stimulus together with a red cross at the bottom of the monitor. Correct answers were followed by 500 ms ISI before the next stimulus (visual or acoustic) was presented.

Each category contained eight different stimuli. For each block, each stimulus was presented twice, resulting in 32 trials for the training blocks and 64 trials for the experimental blocks (8 stimuli x 2 times x 2 categories (x 2 modalities)). Each trial lasted as long as it took for the participant to answer. Mistakes (incorrect categorizations) were signaled by a small red x at the bottom of the screen, forcing the participant to correct his or her mistake. Following each trial there was an inter-stimulus interval (ISI) of 500 ms, during which the screen was black (Fig 4).

#### Data analysis

The data were analyzed following the recommendations by Greenwald and colleagues [67]. In their article, the authors compared a variety of scoring algorithms for the IAT in terms of five criteria: (1) correlations with parallel self-report measures, (2) resistance to an artifact associated with reaction time, (3) internal consistency, (4) sensitivity to known influences on IAT measures, and (5) resistance to known procedural influences. To this end, Greenwald et al. conducted six studies analyzing data from approximately 1.2 million tests. The tested algorithms differed regarding the inclusion or exclusion of data obtained during training trials for the experimental blocks, the setting of an upper- and/or lower-threshold for the reaction time, and the manner in which error trials were handled (i.e., trials in which participants allocated a stimulus not in accordance with the given instructions). As a conclusion of their analysis, the authors suggested an improved scoring algorithm, which was applied in all three experiments reported in the present text. Accordingly, reaction times of error trials were replaced by an error penalty generated from the mean of all correct answers per block plus a 600 ms penalty. However, as the results in all three experiments suggested that reaction times for visual stimuli and for acoustic stimuli differed substantially, the average reaction time was assessed separately per modality of the stimulus.

To test the hypothesis, response latencies were compared between the two experimental conditions. In accordance with the hypothesis, the average response latency for combinations of assumingly associated visual and acoustic stimuli (d1, conforming condition, e.g., pseudo-words with back vowels and pictures with big animals allocated to the same side) was predicted to be shorter than that for combinations of visual and acoustic stimuli that were not believed to be associated (d2, non-conforming condition, e.g., pseudo-words with back vowels allocated to the right side and pictures with big animals to the left) (i.e., ∆d = d2 –d1 > 0).

The effect of the experimental conditions on reaction time latencies was analyzed by applying mixed-effects linear regressions using the lme4 package [68] of the R environment (Version 3.2.3). To test whether the effect of the experimental conditions holds independent of the modality (visual vs. acoustic) or the category (big vs. small for animal pictures and front-vowel vs. back-vowel for pseudo-words) of the stimuli, both factors were included as control variables in the model. All effects as well as the interactions between fixed factors were taken as random at the participant level. To obtain p-values, the car package of the R environment was used by applying type III Wald F tests with Kenward-Roger degrees of freedom approximation [69]. All fixed factors were contrast coded. To ensure normal distribution of the data, a log transformation of the reaction time data was performed. The homogeneity of residuals was validated by inspecting the residuals plotted against fitted values, which yielded no clear indication of heteroscedasticity. Additionally, using aggregated values per participant, paired sample t-tests were conducted in which differences between conditions were tested separately for each stimulus to evaluate the generalizability of the results. Consequently, these post-hoc tests allowed for monitoring the relative influence of the intrinsic qualities of the stimuli on the effect of the experimental condition.

### Results and discussion

The results clearly support the hypothesis. As predicted, mean response latency was significantly shorter in the conforming condition (*M(d1)* = 740.71 ms, *SD* = 151.77 ms) than in the non-conforming condition (*M(d2)* = 858.35 ms, *SD* = 160.90 ms, *F*(1/30.6) = 17.45, *p* < .001). This effect of the experimental conditions on reaction time held for both sensory modalities and for both categories (Table 1).

**Table 1:** Comparison of reaction times in milliseconds per experimental condition separated for stimulus modality and stimulus category.

		conforming		non-conforming	
		mean	SD	mean	SD
**visual stimuli**	**big animals**	682	160	841	223
	**small animals**	669	163	799	181
**acoustic stimuli**	**back vowels**	797	179	853	157
	**front vowels**	814	168	938	236

Next to the effect of the experimental conditions, only stimulus modality exerted a significant effect on the reaction time (*F*(1/34.3) = 34.9, *p* < .001), and the interaction between stimulus modality and experimental conditions showed a tendency towards significance (*F*(1/33.8) = 3.97, *p* < .1). On the other hand, neither stimulus category nor the interaction between stimulus category and experimental conditions had a significant effect on reaction time (*category*: *F*(1/37.5) = 0.70, *p* > .1; condition*category: *F*(1/31.4) = 0.61, *p* > .1).

**Fig 5:**
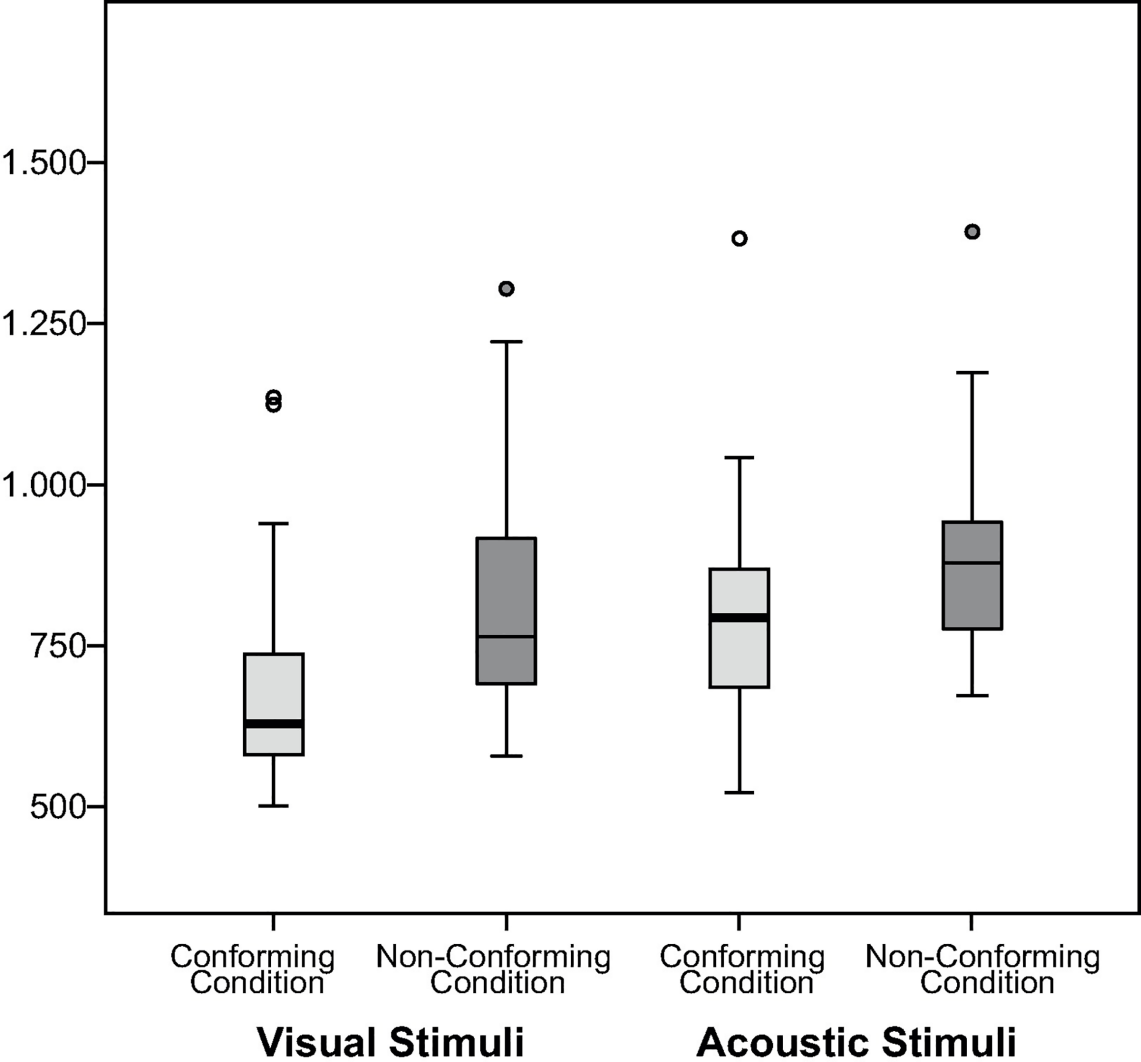
Response latency per experimental block separated by stimulus’ modality for experiment one. The figure contrasts the distribution of reaction-time latencies in milliseconds between experimental blocks (conforming condition to the left in bright gray and non-conforming condition to the right in dark gray), separated by stimulus modality with visual stimuli to the left and acoustic stimuli to the right.

To examine the influence of stimulus modality, the effect of the experimental conditions was analyzed separately for visual and acoustic stimuli. Results of this analysis confirmed that the effect of the experimental conditions was more pronounced for visual than for acoustic stimuli (Fig 5). This difference between visual and acoustic stimuli, however, was to be expected given the nature of the stimuli, with visual stimuli depicting familiar animals and the auditory stimuli consisting of meaningless pseudo-words. Thus, it can be assumed that the distinction between “wild” and “gentle” animals was made automatically and without conscious consideration, whereas the distinction between specific articulatory features of vowels was less intuitive. Still, the difference between the conforming and the non-conforming blocks pointed in the predicted direction for both modalities, with a shorter reaction time in the conforming block than in the non-conforming block (visual: *M(d1)* = 675.78 ms, *SD* = 158.33 ms, *M(d2)* = 820.74, *SD* = 191.71 ms, df = 29, t-value = 3.815, p < .01; acoustic: *M(d1)* = 805.64 ms, *SD* = 165.12 ms, *M(d2)* = 895.97, *SD* = 163.75 ms, df = 29, t-value = 2.372, p < .05).

**Fig 6:**
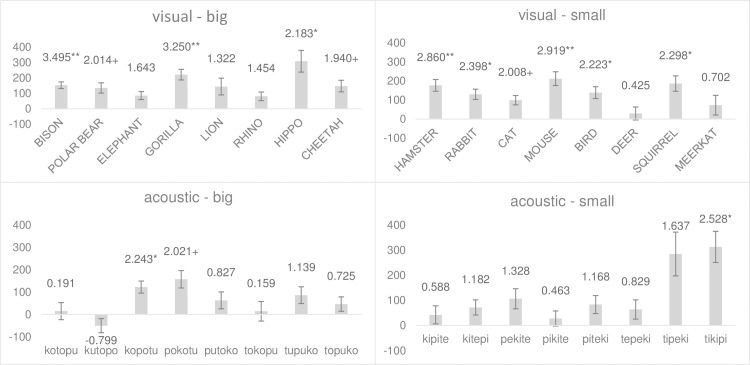
Averaged difference in response latency per stimulus. Bars represent the distance between conforming and non-conforming conditions in milliseconds. Positive values indicate shorter response latencies in the conforming condition. Error bars represent the Standard Error of Mean. Numbers above the bars display t-values. Stars indicate the level of significance, with *** < .001, ** < .01, * < .05, and + < .1. Exact values can be found in the supporting materials (S1 Table).

A post-hoc comparison of reaction time between the experimental conditions conducted separately for each stimulus revealed that all visual stimuli and all but one acoustic stimuli (i.e., kutopo) were categorized faster in the conforming condition than in the non-conforming condition (Fig 6). Note that the results also indicate that phonetic similarity between the name of the visual stimuli and the acoustic stimuli had no relevant effect on participants’ performances. For example, when comparing the results of animals that have only one group of vowels in their Japanese name, it becomes clear that phonetic congruence between visual and acoustic stimuli neither led to a comparatively high (e.g., ELEPHANT, Japanese: zō) nor to a comparatively low (e.g., CHEETAH, Japanese: chītā, HAMSTER, Japanese: hamustā) effect size. Moreover, comparing the results between those animals that contained consonant-vowel combinations of the acoustic stimuli in their Japanese names, again results do not suggest that phonetic congruency had a strong influence on participants’ performance. If similarity between the Japanese names of the animals and the acoustic stimuli had a significant effect, one would expect that on the one hand small animals with consonant + back-vowel combinations (i.e., CAT and BIRD) show a particularly small effect size while big animals with consonant + back-vowel combinations (i.e., POLAR-BEAR) should have a particularly pronounced effect size. However, this prediction is confirmed only for the BIRD but not for the CAT or the POLAR-BEAR, suggesting that congruencies between visual and acoustic stimuli had only a minor effect of the performance of the participants.

Given that the association between pseudo-words and pictures was triggered by the relation between the place of articulation of vowels and the notion of size, one must conclude that the association took place after participants interpreted the content of the pictures. That is, the association between the articulatory features of the verbal stimuli and the visual stimuli took place on a level of cognitive processing, which involved the interpretation of semantic information. Note in particular that animals were not introduced with any reference to size or related concepts but as being either wild or gentle. Thus, the result suggests that the association was not primarily triggered by directly perceptible features, but by semantic conceptualization of these features.

## Experiment II

The aim of the second experiment was to monitor the interaction between perceptible and semantic features of visual stimuli with respect to their association with phonemes. To this end, experiment one was repeated; this time, however, the pictures of big animals were noticeably smaller than the pictures of small animals. Thus, if participants ignore the actual size of the pictures and–as in experiment one–preferably associate back vowels with big animals and front vowels with small animals, it can be concluded that the influence of the content overrides the influence of directly perceptible characteristics on the association between picture and pseudo-word. In other words, the experiment was designed to test the influence of perceptible features (i.e., the size of the pictures) on the association between the vowels and the content of the pictures (i.e., the size of the depicted animals).

### Method

#### Participants

Twenty-six university students (18 female) aged between 18 and 22 (mean 20) volunteered to participate in the experiment. All participants were native Japanese speakers who reported no hearing impairments and had normal or corrected-to-normal vision. Participation was voluntary and participants received a 500-yen gift certificate. All participants gave written informed consent to participate in the study. The experiments were approved by the Ethics Committee of Doshisha University.

#### Materials, apparatus, and procedure

Visual and acoustic stimuli were taken from experiment one. The size of the pictures showing big animals was reduced by one quarter to 11.63 cm x 8.63 cm from originally 15.5 cm x 11.5 cm (height x width). Apparatus, procedure, and data analysis were the same as in experiment one.

### Results and discussion

**Fig 7:**
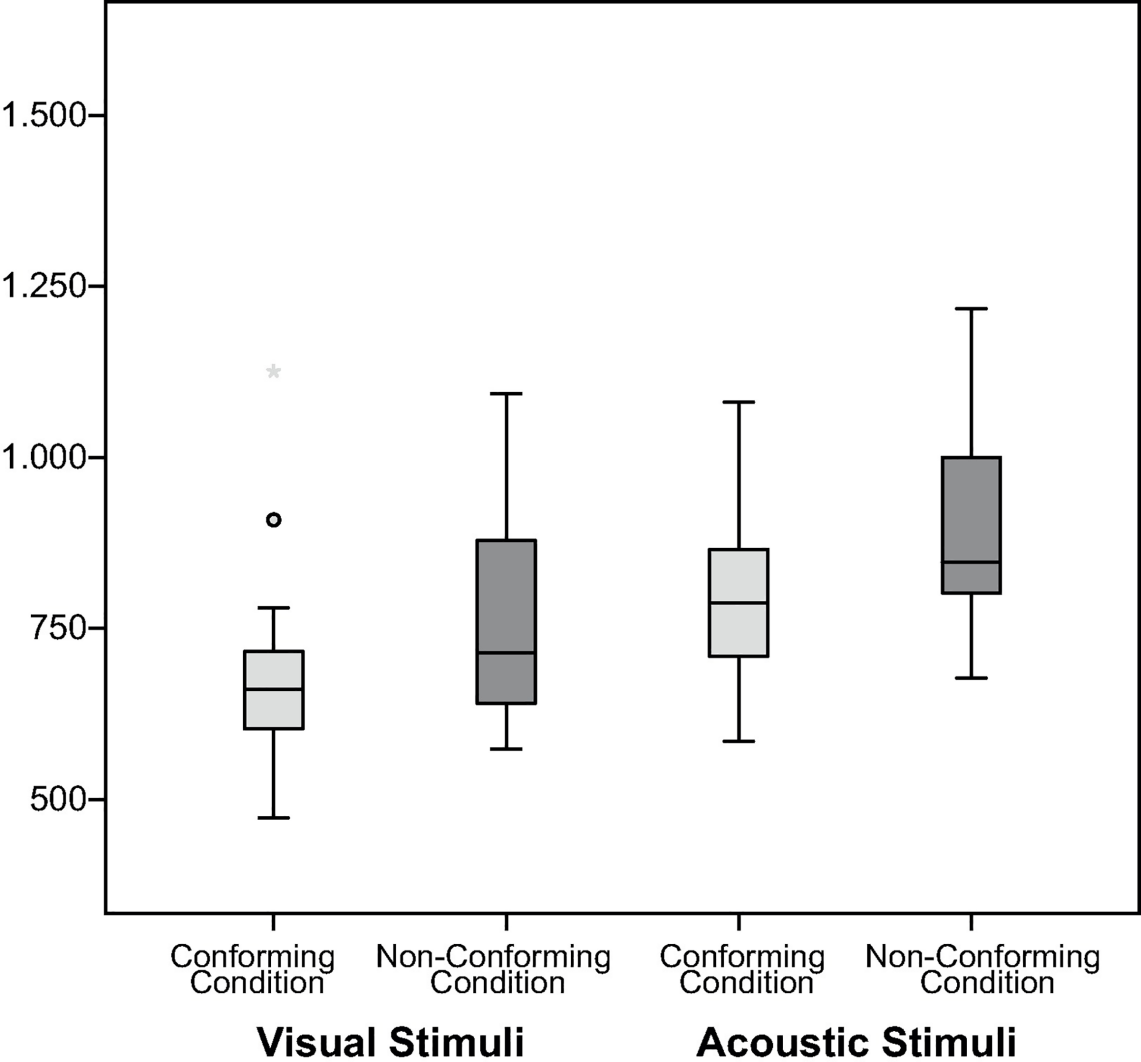
Response latency per experimental block separated by stimulus’ modality for experiment two. The figure contrasts the distribution of response latencies in milliseconds between experimental blocks (conforming condition to the left in bright gray and non-conforming condition to the right in dark gray), separated by stimulus modality with visual stimuli to the left and acoustic stimuli to the right.

As in experiment one, the data clearly confirm the hypothesis. On average, participants answered faster in the conforming condition (*M(d1)* = 746.05 ms, *SD* = 122.03 ms) than in the non-conforming condition (*M(d2)* = 868.35 ms, *SD* = 189.02 ms, *F*(1/28.1) = 13.27, *p* < .01). Next to the effect of the experimental conditions, there was also a significant effect of stimulus modality, which again is most likely attributable to the different degree of familiarity between visual and acoustic stimuli (*F*(1/27.9) = 18.09, *p* < .001). However, there was no significant interaction between experimental conditions and stimulus modality (*F*(1/31.5) = 0.54, *p* < .1), which indicates that the differences between visual and acoustic stimuli did not influence the main effect of the experimental conditions (Fig 7).

Moreover, neither stimulus category nor the interaction between stimulus category and experimental conditions had a significant influence on participants’ reaction time (*category*: *F*(1/43.3) = 0.10, *p* > .1; condition*category: *F*(1/27.1) = 1.56, *p* > .1). In general, a comparison between reaction time latencies between conforming condition and non-conforming condition performed separately for both modalities and both categories showed that the effect of the experimental conditions held across all groups of stimuli (Table 2).

**Table 2:** Comparison of reaction times in milliseconds per experimental condition separated for stimulus modality and stimulus category.

		conforming		non-conforming	
		mean	SD	mean	SD
**visual stimuli**	**big animals**	724	188	819	296
	**small animals**	657	137	811	219
**acoustic stimuli**	**back vowels**	786	158	879	209
	**front vowels**	817	147	965	212

**Fig 8:**
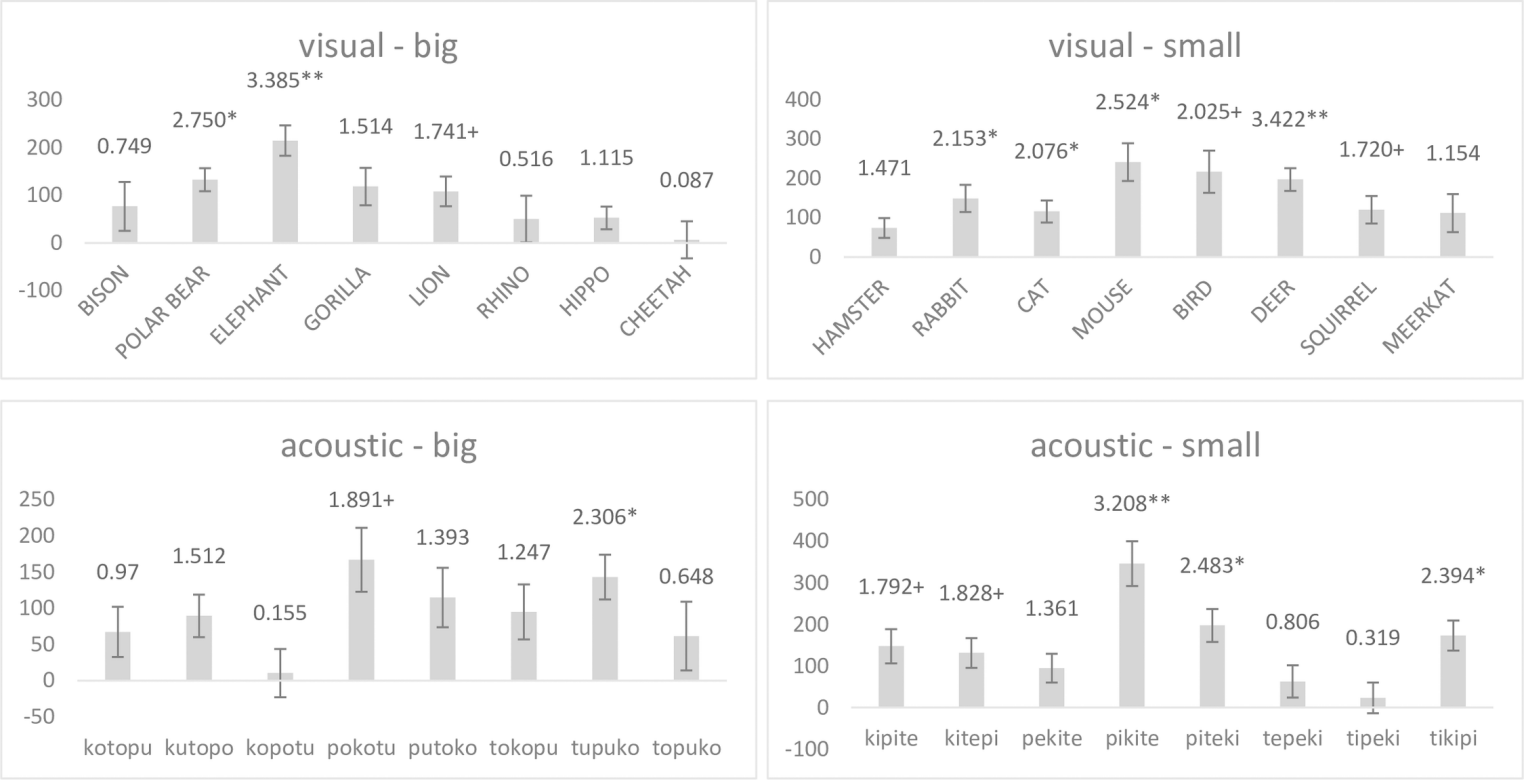
Averaged difference in response latency per stimulus. Bars represent the distance between conforming and non-conforming conditions in milliseconds. Positive values indicate shorter response latencies in the conforming condition. Error bars represent the Standard Error of Mean. Numbers above the bars display t-values. Stars indicate the level of significance, with *** < .001, ** < .01, * < .05, and + < .1. Exact values can be found in the supporting materials (S1 Table).

As in Experiment One, post-hoc analysis again indicates a generalizability of the main effect across all stimuli (Fig 8). However, when comparing the effect per stimulus between the two experiments, it also becomes clear that the difference between conforming condition and non-conforming condition varied markedly. That is, several stimuli that had had a comparatively distinct difference between the two experimental conditions in the first experiment had a comparatively low difference in the second experiment (e.g., GORILLA, HIPPO, HAMSTER for visual stimuli; kopotu and tipeki for acoustic stimuli), and vice versa (e.g., ELEPHANT, BIRD for visual stimuli; tupuko and pikite for acoustic stimuli). This finding strongly suggests that the intrinsic characteristics of the stimuli had only a negligible influence on the results of the experiment. On the other hand, in contrast to Experiment One, there are some indications suggesting a stronger influence of phonetic congruence between the visual and acoustic stimuli on the performance of the participants. That is, for big animals, the one animal with only back vowels (i.e., ELEPHANT) had a relatively wide difference between conforming and non-conforming conditions, whereas the difference was almost zero for the CHEETAH, which is the only big animal with only front-vowels in its Japanese name. However, these two exceptions notwithstanding, phonetic congruence cannot explain the results of all other visual stimuli. For example, the strong effect of the experimental condition on participants’ performance for small animals that contain combinations of plosives with back-vowels equal to those used in the acoustic stimuli (i.e., BIRD and CAT) suggests that cross-modal associations overlay phonetic similarities.

## Experiment III

In the last experiment, visual stimuli depicted emotional body postures. That is, rather than inferring physical properties such as size, the pictures in this experiment were distinguishable on an abstract concept, related to social dominance.

Research suggests that emotional expressions are used by the perceiver as information about the personality or the social role of the expresser (for an overview see [70]). In particular, Knutson [71] reports that fearful expressions are perceived as a sign of low dominance (or subordination), whereas displays of anger promote judgments of high dominance (see also [72, 73]). Though all three studies used facial expressions as stimuli, recent studies imply that the recognition of emotional facial expressions is similar to that of emotional body postures [74, 75]. Thus, in this experiment, eight pictures, half of them displaying fearful body postures (low dominance) and half of them displaying angry body postures (high dominance) were used. Based on the assumption that there is a tendency to relate size with strength and strength with social dominance, it was expected that body postures that express anger are more likely to be associated with back vowels, whereas body postures that express fear are preferably associated with front vowels.

### Method

#### Participants

Twenty-seven university students (16 female) aged between 18 and 25 (mean 20) volunteered to participate in the experiment. All participants were native Japanese speakers who reported no hearing impairments and had normal or corrected-to-normal vision. Participation was voluntary and participants received a 500-yen gift certificate. All participants gave written informed consent to participate in the study. The experiments were approved by the Ethics Committee of Doshisha University.

#### Materials

**Fig 9:**
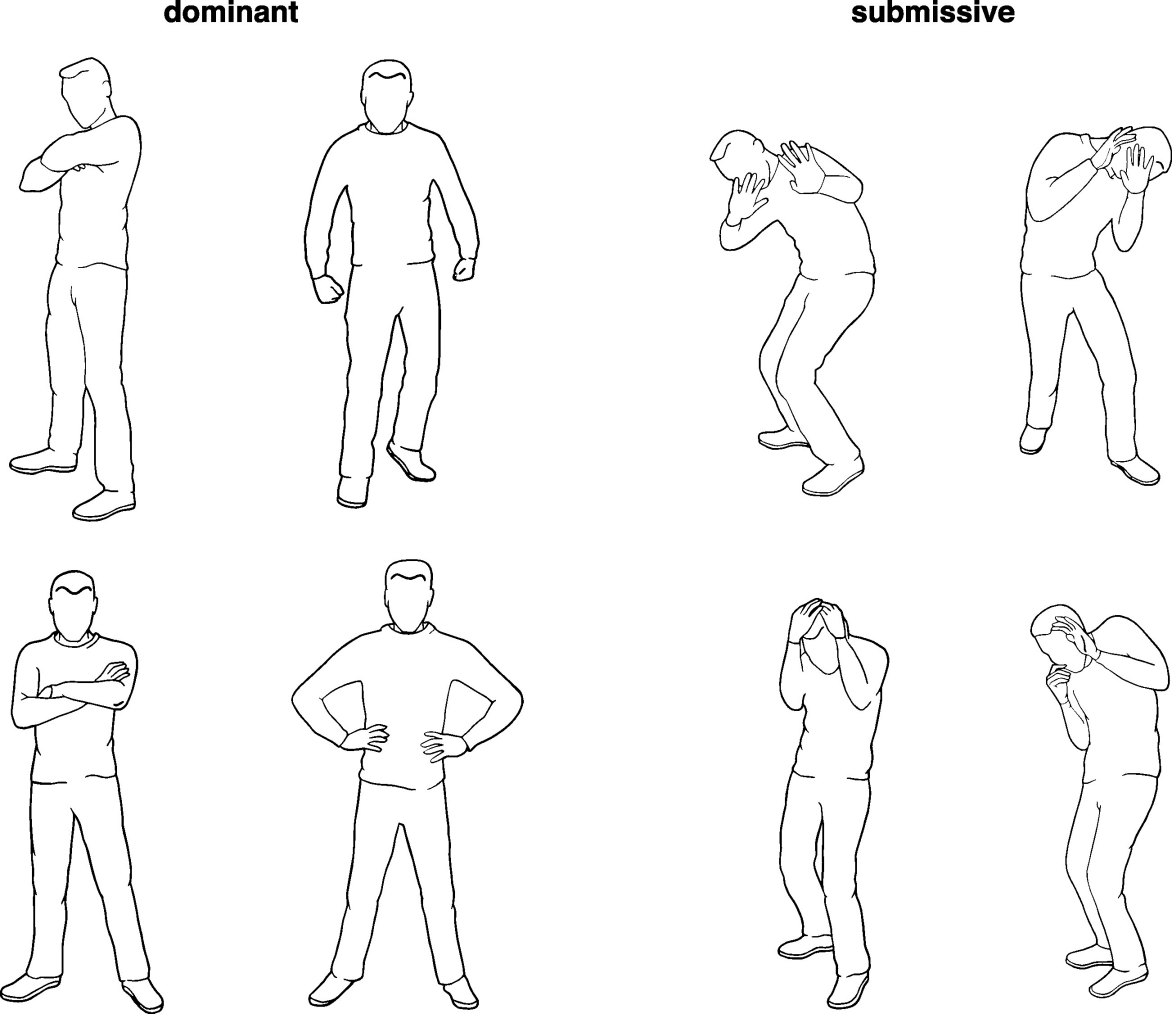
Visual stimuli showing dominant and submissive body postures.

For visual stimuli, eight pictures depicting either dominant or submissive body postures were used (Fig 9). The pictures were drawn by a professional illustrator (NiKo-Illustration). The illustrator was instructed to draw body postures that were either “dominant, aggressive, angry” or “submissive, fearful.” Otherwise the illustrator was uninformed with regard to the goal of the research.

A pretest was conducted to make sure that stimuli were perceived in the intended way. In a paper and pencil test, 51 participants (36 female) aged between 18 and 22 (mean: 19.8) were asked to allocate each individual body posture to one of four basic emotions (i.e., anger, fear, happiness, sadness) [76]. The pre-test was conducted in Japan and all participants were native speakers of Japanese. Both angry and fearful body postures were identified with the respective emotion by a significant majority of participants (anger: 89.3%, χ^2^ = 37.45, p < .001; fear: 83.3%, χ^2^ = 30.74, p < .001). In general, every single stimulus was classified by at least 80% of participants in accordance with the expectation, indicating that the pictures indeed depicted the intended emotion.

Additionally, a second pre-test was conducted to make sure that participants perceive angry and fearful body postures to express high or low dominance, respectively. To this end, 40 participants (25 female, 14 male, one participant gave no answers) aged between 18 and 25 (mean: 19.3) were asked to assess each individual body posture on five bi-polar items. Each item was rated on a 7-point scale (ranging from -3 to +3). The pre-test was conducted in Japan and all participants were native speakers of Japanese. Items were selected to allow the categorization of the emotions expressed by the body posture in a multi-dimensional space comprising valence, arousal, approach, interest, and dominance (Japanese in brackets): contented vs. discontented (満足—不満足), aroused vs. relaxed (興奮—リラックス), affectionate vs. detesting (愛情—嫌悪), inquisitive vs. disinterested (好奇心—無関心), and dominant vs. submissive (支配—服従). The selection of the dimensions followed previous research, according to which an extension of the classical two-dimensional model consisting of *valence* and *arousal* [77–80] to an multi-dimensional model including, for example, *approach*, *attention*, and *dominance* is best suited to render the way emotional expressions are understood [70, 72, 75, 81–85].

Results show that angry pictures were perceived as expressing dominance, whereas fearful body postures were perceived as submissive (mean values for dominant vs. submissive: angry body postures: mean = +1.95, SD = .76; fearful body postures: mean = -1.00, SD = 1.49; t = 7.9, df = 38, p < .001). Other items showed very little difference in effect size and were not significant at the p < .05 level (differences in mean-values of angry vs. fearful body postures with positive values indicate that angry pictures were assessed higher for the respective left attribute): contented vs. discontented: Δmean = .65; inquisitive vs. disinterested: Δmean = .05; aroused vs. relaxed: Δmean = .20). The only exception was affectionate vs. detesting. However, though the difference between angry and fearful body postures was highly significant for this item, both–angry and fearful body postures–were perceived as expressing detesting rather than affection (angry body postures: mean = -1.05, SD = .95; fearful body postures: mean = -2.35, SD = .99; t = 4.25, df = 38, p < .001). Thus, the results of the pretest corroborate previous findings, indicating that angry body postures express high dominance, whereas fearful body postures express low dominance.

Finally, 36 participants (9 female, 26 male, one participant gave no answers) aged between 18 and 22 (mean: 19.6) were asked to assess pictures on differences regarding perceptible features, i.e. their size (big—small—大きい—小さい) and their shape (angular—roundish—角張った—丸っぽい). The pre-test was conducted in Japan and all participants were native speakers of Japanese. As both size and shape of geometrical figures have been found to influence associations with vowels, these last two items were used to make sure that any measured effect is due to semantic (i.e., depicted emotion) and not perceivable (i.e., visual attributes) characteristics of the stimuli. Results revealed no significant differences for size (big vs. small: Δmean = 0.21), but fearful body postures were perceived as significantly more roundish (angry: mean = +2.1, SD = 0.91; fearful: mean = 0.0, SD = 1.86; t = 4.43, df = 34; p < .001).

For acoustic stimuli, eight pseudo-words from experiment one were randomly chosen. These were: kotopu, kutopo, pokotu, tokopu for back vowels and kipite, kitepi, pekite, and pikite for front vowels.

#### Apparatus, procedure, and data analysis

Apparatus, procedure, and data analysis were the same as in experiment one. However, as experiment three employed only eight visual and eight acoustic stimuli (instead of 2*16 as in experiment one and two) every stimulus was presented four times per block (instead of two times) resulting again in 32 trials for training- and 64 trials for experimental-blocks. The instructions for the participants were the same as used in the first two experiments, except for the word for “animal”, which was replaced by “body posture” (人の姿). The visual stimuli for angry and fearful body postures were introduced as “怒った姿” and “恐怖におびえた姿”, respectively.

### Results and discussion

**Fig 10:**
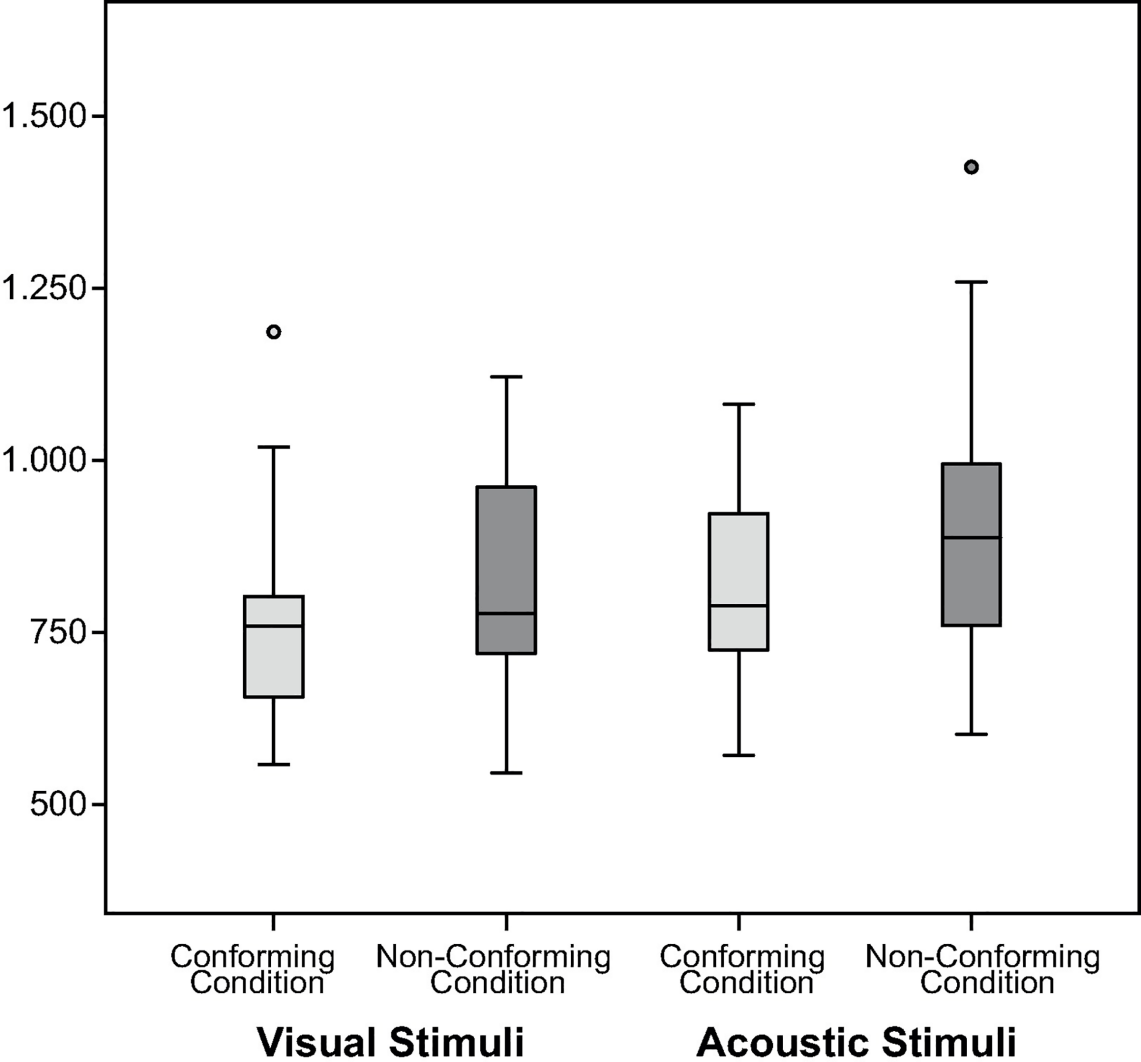
Response latency per experimental block separated by stimulus’ modality for experiment three. The figure contrasts the distribution of reaction time latencies in milliseconds between experimental blocks (conforming condition to the left in bright gray and non-conforming condition to the right in dark gray), separated by stimulus modality with visual stimuli to the left and acoustic stimuli to the right.

In line with the previous experiments, the results again support the hypothesis. On average, participants answered faster in the conforming condition (*M(d1)* = 792.59 ms, *SD* = 134.13 ms) than in the non-conforming condition (*M(d2)* = 867.45 ms, *SD* = 153.85 ms, *F*(1/28.1) = 15.15, *p* < .001). Next to the effect of the experimental conditions, there was again a significant effect of stimulus modality (*F*(1/30.3) = 11.18, *p* < .01), but no significant interaction between experimental conditions and stimulus modality (*F*(1/33.4) = 0.21, *p* > .1). That is, as in the previous two experiments, the influence of stimulus modality on the effect of the experimental conditions did not affect participants’ general tendency to answer faster in the conforming than in the non-conforming condition (Fig 10).

**Fig 11:**
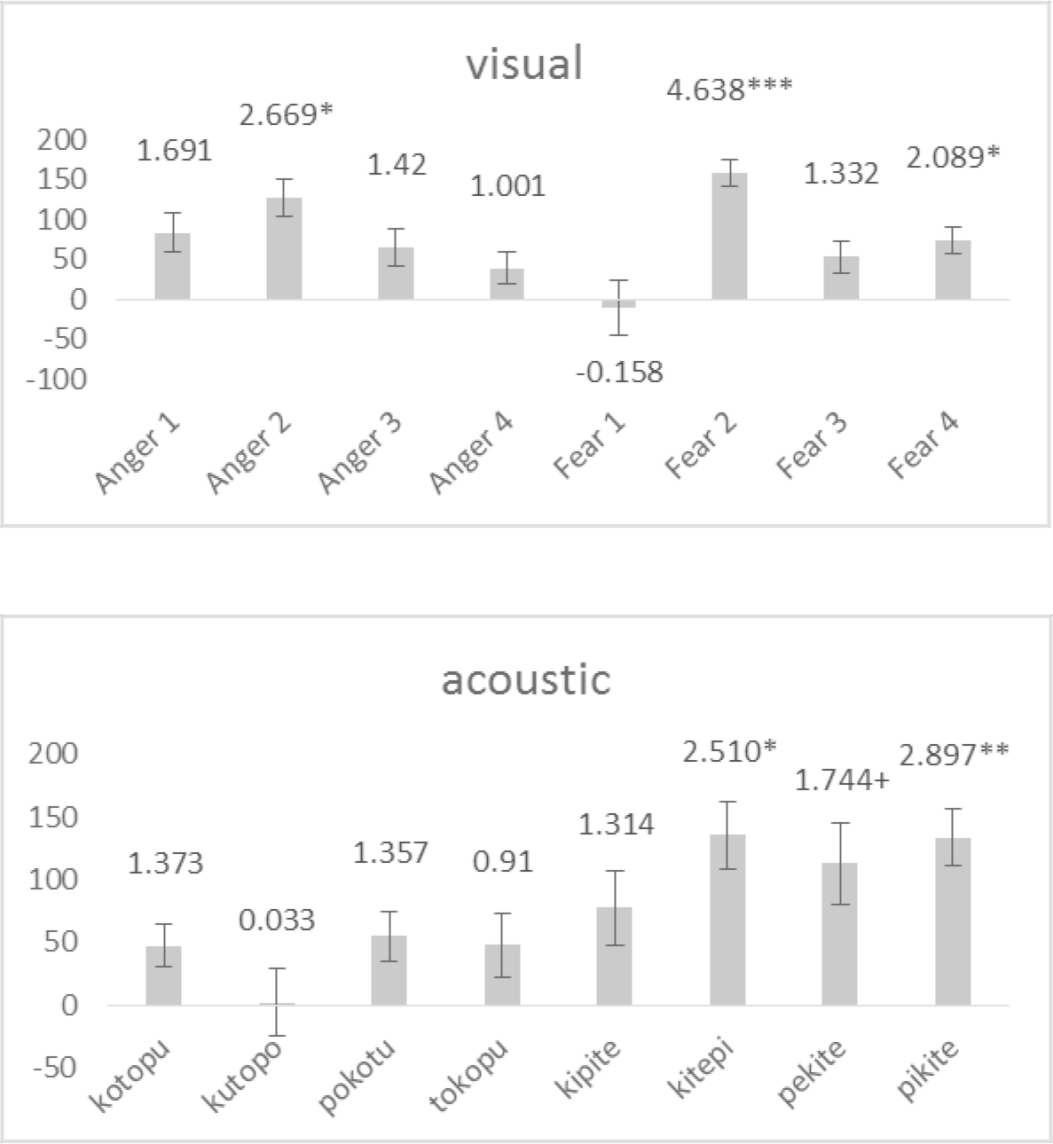
Averaged difference in response latency per stimulus. Bars represent the distance between conforming and non-conforming conditions in milliseconds. Positive values indicate shorter response latencies in the conforming condition. Error bars represent the Standard Error of Mean. Numbers above the bars display t-values. Stars indicate the level of significance, with *** < .001, ** < .01, * < .05, and + < .1. Exact values can be found in the supporting materials (S1 Table).

For stimulus category, neither the factor itself nor its interaction with the experimental conditions proved significant (*category*: *F*(1/32.8) = 0.06, *p* > .1; category*condition: *F*(1/32.4) = 1.30, *p* > .1). In general, a comparison of reaction time latencies between the experimental blocks separated for stimulus modality and stimulus category confirmed the same trend for all sub-groups of stimuli (Table 3). Moreover, comparing the response latency between conforming and non-conforming conditions separately per stimulus reveals that the results confirm the hypothesis (Fig 11) with only one exception, suggesting that differences in the participants’ performance was dominantly influenced by the experimental condition whereas intrinsic qualities of the stimuli had only a moderate effect.

**Table 3:** Comparison of reaction times in milliseconds per experimental condition separated for stimulus modality and stimulus category.

		conforming		non-conforming	
		mean	SD	mean	SD
**visual stimuli**	**big animals**	757	158	836	189
	**small animals**	764	164	832	151
**acoustic stimuli**	**back vowels**	836	148	874	163
	**front vowels**	813	165	928	255

That is, visual stimuli depicting anger were associated with pseudo-words containing back vowels, while visual stimuli showing fear were associated with pseudo-words containing front vowels. As shown in previous studies [71, 73] and as suggested by the results of the pre-test outlined above, angry body postures resembled the notion of high social dominance whereas fearful body postures were related to low social dominance. Consequently, the results corroborate the hypothesis, suggesting that the place of articulation of vowels is not only related to physical size or strength, but also to social hierarchy.

However, it has to be noted that complex semantic stimuli always vary along several features, which are difficult to control. In this experiment, a pre-test was conducted in an attempt to control influences exerted by the shape of the pictures. This pre-test revealed that angry body postures were perceived to be more angular and fearful pictures more roundish. As the used back vowels are articulated with rounded lips and as previous research revealed that such articulatory movements foster associations with roundish shapes [86, 87], the assessment of the shape of the pictures would have predicted an outcome opposing the results of this experiment. Thus, as in experiment two, the results suggest that semantic features of the pictures dominated the association with the pseudo-words, whereas influences from perceptible features were overridden. Considered together, the results of all three experiments clearly suggest that found associations are attributable to the interpretation of the pictures as depicting physical or social dominance.

## General discussion

The aim of this paper was to assess to what extent articulatory-acoustic features of phonemes show implicit associations with semantic stimuli. Based on the results of previous studies showing that front vowels are preferably associated with stimuli that refer to smallness and back vowels with stimuli that refer to bigness, it was hypothesized that the place of articulation (front vs. back) also predicts the association of vowels with pictures implying physical or social dominance. Results of the experiments support the hypothesis: pseudo-words containing back vowels were associated with pictures depicting big animals or dominant behavior and pseudo-words containing front vowels were associated with pictures depicting small animals and submissive behavior. This association between the articulatory-acoustic characteristics of phonemes and the content of the pictures seems independent of directly perceptible features of the pictures themselves, as the size and shape of the used pictures would have predicted the opposite results. In other words, the influence of the semantic features of the pictures outweighed the perceptible features in triggering the association.

To the best of my knowledge, this was the first study in which articulatory-acoustic features of phonemes were tested for implicit associations with semantic stimuli that referred to the concept of dominance through their content. In contrast, previous studies used stimuli that directly referred to the concept of size [40, 41] or to related concepts, such as weight or strength [42–46].

The results reported here, therefore, go beyond the well-established association between the place of articulation and the notion of size, suggesting that phonemes–at least potentially–elicit highly abstract semantic connotations. These connotations seem to be implicit, considering the settings of the presently described experiments. However, it has to be noted that the term implicit is not well defined and, thus, requires further clarification as to what level of cognitive processing it refers to [88]. Moreover, it has been questioned to what extent the associations assessed by the Implicit Association Test (which was applied in this study) are actually implicit [89]. Another problem concerns the nature of the stimuli. Complex semantic stimuli vary along various dimensions, which all might have had an influence on the results. Furthermore, the labelling of the stimuli as “wild” vs. “gentle” or “angry” vs. “fearful” for the visual stimuli and as “bright” vs. “dark” for the acoustic stimuli might have influenced the results as well. To the best of my knowledge, there is no evidence that the labels used for the visual stimuli are associated with those used for the acoustic stimuli.

Moreover, in addition to associations based on semantic relations, another undesired effect can arise from phonetic similarities between the labels used to introduce the stimuli to the participants and the pseudo-words. For the first two experiments, the distinction between “wild” (Japanese “獰猛”, read “dōmō”) and “gentle” (Japanese “温和”, read “onwa”) guaranteed that both labels contain back-vowels but no front-vowels. A possible interference, however, could be attributed to the labels used for the visual stimuli in the third experiment as the Japanese word for ‘anger’ (怒った姿, read “okottasugata”) contains only back-vowels, whereas the word for ‘fear’ (恐怖におびえた姿, read “kyōfunniobietakatta”) also contains front-vowels. Though it seems to be unlikely that participants recalled the label of the category of a presented stimulus instead of focusing on attributes related to the stimulus itself when performing a speeded classification task, whether this difference in labelling had an influence on the performance of the participants cannot be determined.

More serious concerns, however, arise from potential confounding variables related to the names of the depicted animals in the first two experiments. Consequently, visual stimuli were carefully selected to avoid undesired effects due to phonetic congruence between the name of the depicted animal and the characteristics of acoustic stimuli. Moreover, a comparison of the results between the first and the second experiment clearly suggests that phonetic characteristics had no critical influence on the performance of the participants. First, all visual stimuli–independent of their appearance and the names of the depicted animals–had shorter response latencies in the conforming condition than in the non-conforming condition. Second, the relative differences in response latency between conforming and non-conforming conditions per stimulus varied markedly between the experiments, in that several pictures with relatively high differences in the first experiment had relatively low differences in the second experiment, and vice versa. And finally, phonetic similarities between the Japanese names of depicted animals and the distinctive characteristics of the acoustic stimuli did not lead to a better or worse performance of the participants. These findings suggest that intrinsic qualities had only a marginal influence on the results.

However, though these observations strongly imply that the differences in response latency can mainly be attributed to the experimental conditions, influences from confounding variables ultimately cannot be excluded, especially because it is unclear which attributes would actually cause associations across sensory modalities. Regarding the names of the depicted animals, for example, it is not clear to what extent the general occurrence of front-vowels and back-vowels, their ratio, their combination with consonants, or their position within a word exerts an influence on cross-modal associations. Thus, in order to control for the undesired effects of potential co-variates, further studies that replicate the results using different research designs and stimuli are necessary.

In summary, reservations due to possible confounding variables notwithstanding, the results reported here strongly indicate that associations between phonemes and visual characteristics–such as shape and size–do not stop at a perceptual level but also include a conceptual level of cognitive processing. Given the nature of the visual stimuli used here and the results of previous studies [44, 45, 50], it seems fair to conclude that vowels, due to their articulatory-acoustic features, are not only related to size, but to an abstract and amodal concept of magnitude, conveying a sense of *more*, *bigger*, *heavier*, *louder*, *stronger*, etc. Following this idea, both big objects and dark sounds are allocated to the same pole of the magnitude dimension, causing their cross-modal association. Such a hypothesis has previously been formulated by Martino and Marks [90], who proposed that cross-modal “congruence effects reflect the coding or recoding of perceptual information into a common, abstract semantic representation that captures the synesthetic relation between dimensions” (p. 747). Additionally, I argue that the relation between dark sounds and the concept of bigness also has an emotional component. Objects perceived as big and strong most likely have an intimidating effect, eliciting feelings like respect or even fear. Thus, on this basis, acoustic characteristics of phonemes can also be associated with social behavior, perceiving dominant and aggressive behavior as potentially harmful and, therefore, related to dark sounds.

Though this interpretation of the results is admittedly speculative, it suggests that further endeavors for a better understanding of sound iconicity might also give insights in cognitive processes generally involved in language processing. To give one example, a potential question arising from this research concerns the influence of the phonetic structure of texts on their aesthetic perception. Inspired by Jakobson’s [91] accentuation of the role linguistic form plays in poetic language, theoretical [92] and empirical works [20, 93–96] have recently investigated the effect of structural elements of language on aesthetic evaluation, looking for example at readers’ appreciation of meter, rhyme, syntactic features, or the phonetic structure of a text. Thus, in the light of the results presented here, this line of research can be extended, for example by exploring the effect of semantic congruencies between content and phonetic structure of texts.

## Supporting information

S1 Table Tables with averaged difference in response latency per stimulus for all three experiments. (DOCX)

S1 Text Instructions. Example of instructions for participants with English translation. Note: Order of the experimental blocks (first conforming and second non-conforming or vice versa) and allocation of stimuli (e.g., big animals to the right or to the left in conforming block) was randomized.
(DOCX)

S1 Dataset Implicit association test. Folder contains text files with original data of IAT experiments. Each text file represents data from one participant. Together with the data the folder also contains a ‘read me’ file that explains how to read the files.
(ZIP)

S2 Dataset Pre-tests. Folder contains data of three pre-tests (Experiment 3). In three questionnaires participants were asked to allocate visual stimuli to one of four basic emotions (pre-test 1), to categorize the emotions expressed by the stimuli (pre-test 2), and to assess the shape and size of the stimuli (pre-test 3).
(ZIP)
