# Supplementary material for: Sound iconicity of abstract concepts: Place of articulation is implicitly associated with abstract concepts of size and social dominance
Source: PLoS One. 2017 Nov 1;12(11):e0187196. doi: 10.1371/journal.pone.0187196 (PMC5665516; doi:10.1371/journal.pone.0187196)
Supplement: S1 Table — (DOCX) [file pone.0187196.s001.docx]

**S3: Tables with averaged differences in response latency per stimulus.** Mean-values display differences between experimental conditions in milliseconds. Positive values indicate shorter response latencies in the conforming condition. Stars indicate the level of significance, with ^+^ < .1, * < .05, ** < .01, *** < .001. Aggregated values per participant were used for the test.

**Experiment I: Visual stimuli**

| **lfd** | **Stimulus** | **Japanese** | **Mean** | **SE** | **df** | **t-value** |
| --- | --- | --- | --- | --- | --- | --- |
| 1 | BISON | ウシ | 152.77 | 43.71 | 29 | 3.495** |
| 2 | POLAR BEAR | シロクマ | 135.19 | 67.12 | 29 | 2.014^+^ |
| 3 | ELEPHANT | ゾウ | 85.19 | 51.84 | 29 | 1.643 |
| 4 | GORILLA | ゴリラ | 221.22 | 68.08 | 29 | 3.250** |
| 5 | LION | ライオン | 144.25 | 109.09 | 29 | 1.322 |
| 6 | RHINO | サイ | 81.02 | 55.71 | 29 | 1.454 |
| 7 | HIPPO | カバ | 308.50 | 141.35 | 29 | 2.183* |
| 8 | CHEETAH | チーター | 147.14 | 75.86 | 29 | 1.940^+^ |

| **lfd** | **Stimulus** | **Japanese** | **Mean** | **SE** | **df** | **t-value** |
| --- | --- | --- | --- | --- | --- | --- |
| 1 | HAMSTER | ハムスター | 176.77 | 61.81 | 29 | 2.860** |
| 2 | RABBIT | ウサギ | 129.86 | 54.16 | 29 | 2.398* |
| 3 | CAT | ネコ | 98.79 | 49.20 | 29 | 2.008^+^ |
| 4 | MOUSE | ネズミ | 212.18 | 72.69 | 29 | 2.919** |
| 5 | BIRD | トリ | 138.72 | 62.41 | 29 | 2.223* |
| 6 | DEER | シカ | 29.05 | 68.42 | 29 | .425 |
| 7 | SQUIRREL | リス | 186.13 | 81.01 | 29 | 2.298* |
| 8 | MEERKAT | ミーアキャット | 72.57 | 103.34 | 29 | .702 |

**Experiment I: Acoustic stimuli**

| **lfd** | **Stimulus** | **Japanese** | **Mean** | **SE** | **df** | **t-value** |
| --- | --- | --- | --- | --- | --- | --- |
| 1 | kotopu | コトプ | 14.53 | 76.12 | 29 | .191 |
| 2 | kutopo | クトポ | -50.51 | 63.21 | 29 | -.799 |
| 3 | kopotu | コプテュ | 122.34 | 54.55 | 29 | 2.243* |
| 4 | pokotu | ポトク | 157.25 | 77.80 | 29 | 2.021^+^ |
| 5 | putoko | プトコ | 62.72 | 75.88 | 29 | .827 |
| 6 | tokopu | トコプ | 13.95 | 87.76 | 29 | .159 |
| 7 | tupuko | テュプコ | 86.13 | 75.63 | 29 | 1.139 |
| 8 | topuko | トプコ | 46.19 | 63.71 | 29 | .725 |

| **lfd** | **Stimulus** | **Japanese** | **Mean** | **SE** | **df** | **tvalue** |
| --- | --- | --- | --- | --- | --- | --- |
| 1 | kipite | キピテ | 42.35 | 72.00 | 29 | .588 |
| 2 | kitepi | キテピ | 71.65 | 60.64 | 29 | 1.182 |
| 3 | pekite | ペキテ | 106.34 | 80.05 | 29 | 1.328 |
| 4 | pikite | ピキテ | 27.73 | 59.85 | 29 | .463 |
| 5 | piteki | ピテキ | 83.50 | 71.46 | 29 | 1.168 |
| 6 | tepeki | テペキ | 63.64 | 76.79 | 29 | .829 |
| 7 | tipeki | ティぺキ | 284.59 | 173.90 | 29 | 1.637 |
| 8 | tikipi | ティキピ | 312.91 | 123.78 | 29 | 2.528* |

**Experiment II: Visual stimuli**

| **lfd** | **Stimulus** | **Mean** | **SE** | **df** | **t-value** |
| --- | --- | --- | --- | --- | --- |
| 1 | BISON | 76.93 | 102.65 | 24 | .749 |
| 2 | BEAR | 132.40 | 48.14 | 24 | 2.750* |
| 3 | ELEPHANT | 214.64 | 63.40 | 24 | 3.385** |
| 4 | GORILLA | 118.40 | 78.21 | 24 | 1.514 |
| 5 | LION | 108.13 | 62.11 | 24 | 1.741^+^ |
| 6 | RHINO | 50.50 | 97.82 | 24 | .516 |
| 7 | HIPPO | 52.65 | 47.20 | 24 | 1.115 |
| 8 | CHEETAH | 6.70 | 77.49 | 24 | .087 |

| **lfd** | **Stimulus** | **Mean** | **SE** | **df** | **t-value** |
| --- | --- | --- | --- | --- | --- |
| 1 | HAMSTER | 74.32 | 50.52 | 24 | 1.471 |
| 2 | RABBIT | 148.92 | 69.18 | 24 | 2.153* |
| 3 | CAT | 116.19 | 55.95 | 24 | 2.076* |
| 4 | MOUSE | 241.81 | 95.79 | 24 | 2.524* |
| 5 | BIRD | 217.05 | 107.17 | 24 | 2.025^+^ |
| 6 | DEER | 197.62 | 57.74 | 24 | 3.422** |
| 7 | SQUIRREL | 120.63 | 70.15 | 24 | 1.720^+^ |
| 8 | MEERKAT | 112.06 | 97.11 | 24 | 1.154 |

**Experiment II: Acoustic stimuli**

| **lfd** | **Stimulus** | **Mean** | **SE** | **df** | **t-value** |
| --- | --- | --- | --- | --- | --- |
| 1 | kotopu | 67.19 | 69.26 | 24 | .970 |
| 2 | kutopo | 89.39 | 59.11 | 24 | 1.512 |
| 3 | kopotu | 10.37 | 66.75 | 24 | .155 |
| 4 | pokotu | 167.05 | 88.33 | 24 | 1.891^+^ |
| 5 | putoko | 114.64 | 82.30 | 24 | 1.393 |
| 6 | tokopu | 94.79 | 76.02 | 24 | 1.247 |
| 7 | tupuko | 143.01 | 62.00 | 24 | 2.306* |
| 8 | topuko | 61.42 | 94.78 | 24 | .648 |

| **lfd** | **Stimulus** | **Mean** | **SE** | **df** | **t-value** |
| --- | --- | --- | --- | --- | --- |
| 1 | kipite | 147.52 | 82.31 | 24 | 1.792^+^ |
| 2 | kitepi | 131.47 | 71.90 | 24 | 1.828^+^ |
| 3 | pekite | 94.86 | 69.71 | 24 | 1.361 |
| 4 | pikite | 346.03 | 107.86 | 24 | 3.208** |
| 5 | piteki | 197.62 | 79.58 | 24 | 2.483* |
| 6 | tepeki | 62.86 | 78.02 | 24 | .806 |
| 7 | tipeki | 23.50 | 73.71 | 24 | .319 |
| 8 | tikipi | 173.04 | 72.28 | 24 | 2.394* |

**Experiment III: Visual stimuli**

| **lfd** | **Stimulus** | **Mean** | **SE** | **df** | **t-value** |
| --- | --- | --- | --- | --- | --- |
| 1 | Anger 1 | 83.35 | 49.28 | 26 | 1.691 |
| 2 | Anger 2 | 126.32 | 47.34 | 26 | 2.669* |
| 3 | Anger 3 | 65.20 | 45.91 | 26 | 1.420 |
| 4 | Anger 4 | 38.86 | 38.83 | 26 | 1.001 |
| 5 | Fear 1 | -10.60 | 67.28 | 26 | -.158 |
| 6 | Fear 2 | 157.57 | 33.97 | 26 | 4.638*** |
| 7 | Fear 3 | 52.85 | 39.67 | 26 | 1.332 |
| 8 | Fear 4 | 73.73 | 35.30 | 26 | 2.089* |

**Experiment III: Acoustic stimuli**

| **lfd** | **Stimulus** | **Mean** | **SE** | **df** | **t-value** |
| --- | --- | --- | --- | --- | --- |
| 1 | kotopu | 47.40 | 34.51 | 26 | 1.373 |
| 2 | kutopo | 1.80 | 54.02 | 26 | .033 |
| 3 | pokotu | 54.61 | 40.26 | 26 | 1.357 |
| 4 | tokopu | 47.49 | 52.21 | 26 | .910 |
| 5 | kipite | 77.24 | 58.77 | 26 | 1.314 |
| 6 | kitepi | 135.39 | 53.93 | 26 | 2.510* |
| 7 | pekite | 112.76 | 64.65 | 26 | 1.744^+^ |
| 8 | pikite | 133.70 | 46.16 | 26 | 2.897** |
