# Supplementary material for: Sound iconicity of abstract concepts: Place of articulation is implicitly associated with abstract concepts of size and social dominance
Source: PLoS One. 2017 Nov 1;12(11):e0187196. doi: 10.1371/journal.pone.0187196 (PMC5665516; doi:10.1371/journal.pone.0187196)
Supplement: S1 Text — Example of instructions for participants with English translation. Note: Order of the experimental blocks (first conforming and second non-conforming or vice versa) and allocation of stimuli (e.g., big animals to the right or to the left in conforming block) was randomized. (DOCX) [file pone.0187196.s002.docx]

**S 1: Instructions**

Page 1: Introduction (English translation below)

| 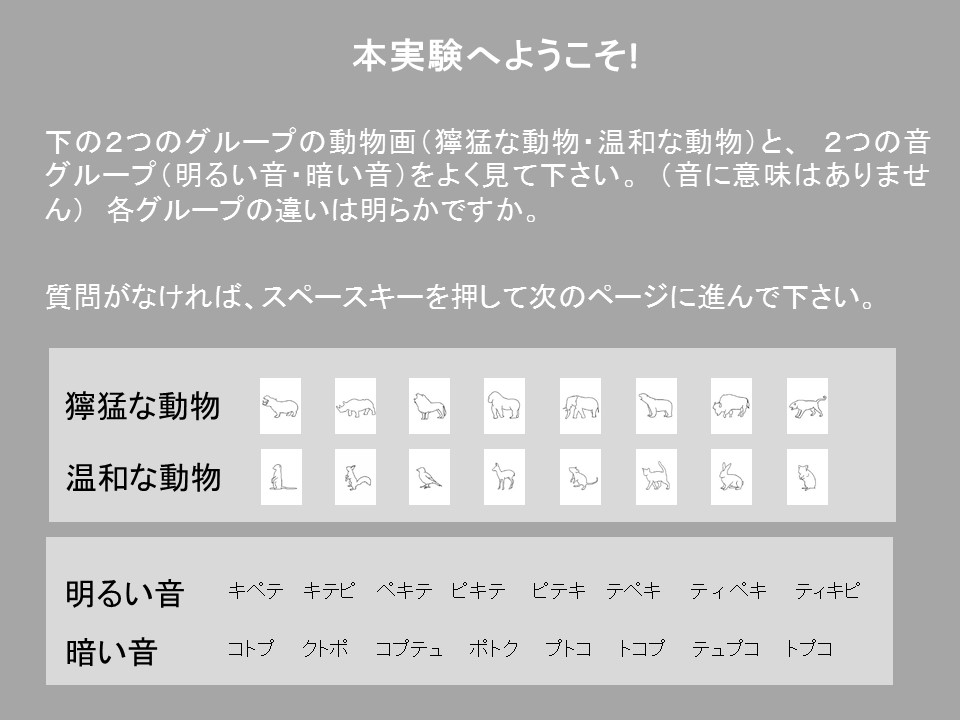 |
| --- |

English translation:

Welcome to the experiment!

Please look carefully at the two groups of animal pictures (wild animals・calm animals) and two groups of words (bright sounds・dark sounds) (Note that the words do not make sense). Make sure you understand the difference between each group.

If you do not have any questions press the space-bar to continue to the next page.

Page 2: Instructions (English translation below)

| 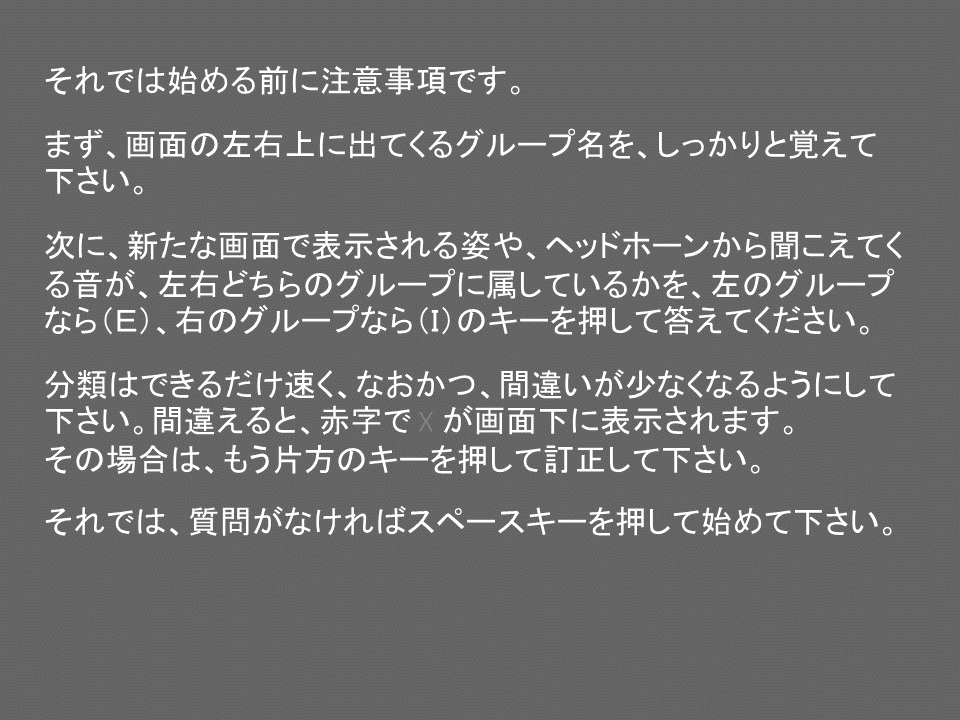 |
| --- |

Before we start please read the following introduction.

First, on the upper right and upper left side you will see which group of stimuli is allocated to which side. Please pay careful attention to this allocation.

Next, you will either see a picture on the screen or you will hear a sound through the headphones. Depending on which side the picture or sound belongs, you should press the E-key for left or the I-key for right, respectively. Please answer as quickly and correctly as possible. When you make a mistake, there will be a red X at the bottom of the screen. In this case, please correct your answer.

If you have no questions, please press the space bar to start.

Page 3: Example for a beginning of a training block (English translation below).

| 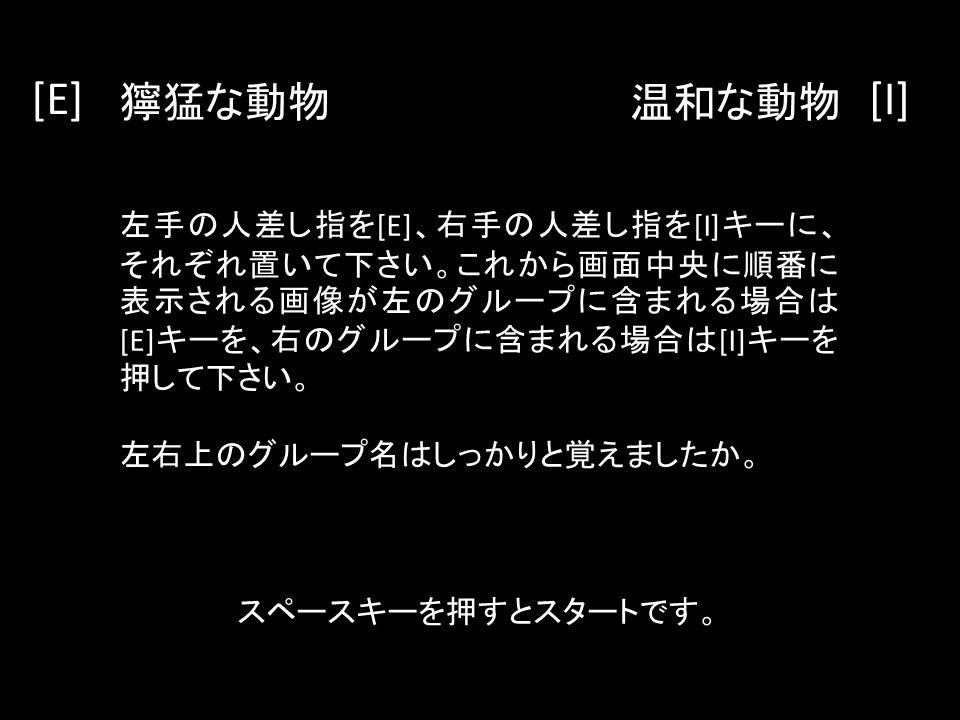 |
| --- |

Upper left corner: wild animals

Upper right corner: calm animals

Please position your left index finger on the E-key and your right index finger on the I-key. In the following you will see one picture after another in the middle of the screen. If the picture belongs to the left group press the E-key, if it belongs to the right group press the I-key.

Please look carefully, which group belongs to the left side and which belongs to the right side.

Press the space bar to start the experiment.
